# Case Report: Life-threatening acute subdural hematoma associated with human parvovirus B19 infection in a young adult

**DOI:** 10.3389/fsurg.2026.1762977

**Published:** 2026-02-25

**Authors:** Na Wang, Yanfei Li, Minghui Zhang, Xin Li, Xin Guan

**Affiliations:** Department of Neurosurgery, Xuanwu Hospital, Capital Medical University, Beijing, China

**Keywords:** acute subdural hematoma, human parvovirus B19, infection control, intracranial hypertension, neurocritical nursing, postoperative management, ultra-early rehabilitation

## Abstract

Severe acute subdural hematoma (ASDH) secondary to human parvovirus B19 (B19V) infection is exceptionally rare in adults and presents unique neurocritical care challenges. We report a previously healthy 35-year-old woman with a history of two cesarean sections and no chronic diseases, who developed a sudden headache followed by rapid neurological deterioration. Head computed tomography (CT) revealed a massive right fronto-temporo-parietal ASDH with marked midline shift. Notably, one week prior to admission, she traveled to Guangzhou with her children, two of whom developed fever three days and confirmed B19V infection before her symptoms, suggesting possible household transmission. The association between ASDH and B19V infection was established by excluding other common causes (such as trauma, vascular malformations, coagulopathies, and other infections) through detailed history, imaging, and laboratory investigations. The association between ASDH and B19V infection was established by: (1) exclusion of other common etiologies (trauma, vascular malformations, coagulopathies, and other infections) through detailed history, imaging, and laboratory investigations; (2) confirmation of acute B19V infection by serological and molecular testing; (3) clear temporal relationship with household exposure. Emergency hematoma evacuation and decompressive craniectomy were performed. Specialized neurocritical nursing included automated quantitative pupillometry for early detection of intracranial hypertension, and strict hemodynamic control, with continuous arterial blood pressure monitoring and antihypertensive therapy to maintain systolic blood pressure within 110–130mmHg, minimizing the risk of rebleeding and further neurological injury. Ultra-early rehabilitation was initiated. Immediate postoperative extubation was deferred due to the timing of surgery (early morning), limited night staffing, and the need for close postoperative neurological and respiratory monitoring. The patient was safely weaned from mechanical ventilation on postoperative day 1 after confirming stable neurological and respiratory function. Muscle strength was evaluated using the manual muscle testing (MMT) scale, which improved to grade IV in all limbs. She was transferred for rehabilitation on postoperative day 23. To our knowledge, this represents one of the first reports of favorable neurological recovery in an adult with life-threatening B19V-associated intracranial hemorrhage. This case demonstrates that rapid surgical intervention combined with monitoring, targeted infection control, and intensive early rehabilitation can achieve favorable outcomes in this exceedingly rare condition.

## Introduction

Severe acute subdural hemorrhage (ASDH) is a life-threatening intracranial bleed, most commonly following head trauma, but it can also occur spontaneously in the context of coagulopathy or vascular pathology (1). Mortality approaches 40%–60% and only 19%–45% of survivors achieve meaningful neurological recovery (2, 3). Surgical evacuation remains the cornerstone of treatment for ASDH patients with intracranial hypertension or significant mass effect; however, the perioperative period is characterized by complex pathophysiological changes and a high risk of secondary brain injury (4, 5). Favorable outcomes therefore depend not only on timely surgical decompression but also on specialized neurocritical care. Although virus-associated intracranial bleeding is uncommon and the available evidence is largely limited to case reports and small series, a growing body of literature suggests that several viral infections may be complicated by intracerebral or subdural hemorrhage. Reported examples include Herpes Simplex Virus (HSV), dengue, arboviruses, and, more recently, SARS-CoV-2 infection (6).

Human parvovirus B19 (B19V) is a non-enveloped, single-stranded DNA virus of the genus Erythrovirus and is a common pathogen, particularly in children (7–9). Transmission occurs primarily via respiratory secretions and exposure to infected blood (10). While infection is often self-limited in immunocompetent hosts, B19V can cause significant hematological complications, such as aplastic crisis, chronic anemia, and thrombocytopenia, by suppressing erythropoiesis or triggering immune-mediated vascular injury (11). B19V-associated intracranial hemorrhage has been described in fetuses; adult cases are exceptionally rare (12, 13). While a causal association between B19V and intracranial hemorrhage has not been demonstrated, previous imaging studies have shown dilation of cerebral vessels in the frontal and occipital lobes among infected patients (14). This vasodilation may compromise vascular integrity and thereby increase the risk of intracranial hemorrhage.

In May 2024, we managed a previously healthy 35-year-old woman with severe ASDH temporally associated with acute B19V infection. The case involved multiple challenges, including anemia, high risk of rebleeding, strict infection prevention and control, and the design of an ultra-early rehabilitation strategy after decompressive craniectomy. Through a multidisciplinary neurocritical care approach, the patient achieved a favorable neurological outcome. In this report, we summarize our nursing management in four key domains: multimodal intracranial pressure (ICP) monitoring (including automated quantitative pupillometry), comprehensive postoperative care, targeted infection control for B19V, and ultra-early neurological rehabilitation. The aim is to provide a reference for managing similar complex cases.

## Case description

A 35-year-old woman presented to the emergency department on May 13, 2024, with a sudden headache, dizziness, and emesis, who is a company employee. The patient family reported no known chronic medical conditions, no prior neurologic history, no long-term medication use, no alcohol or illicit drug use, no family history, and no recent use of antiplatelet or anticoagulant agents, and had undergone two cesarean deliveries (2018 and 2020). Denied any head or neck trauma preceding symptom onset. Socially, she lived with her family and had close household contact with her children. One week before the presentation, she traveled with her children to Guangzhou, a high-risk area for infectious diseases in China. Two children developed fever approximately three days before their symptom onset. Given that human parvovirus B19 can spread through respiratory droplets and close contact within households, this exposure history was considered relevant. Diagnoses were right acute subdural hematoma, acute human parvovirus B19 infection, and moderate anemia.

**Figure 1:**
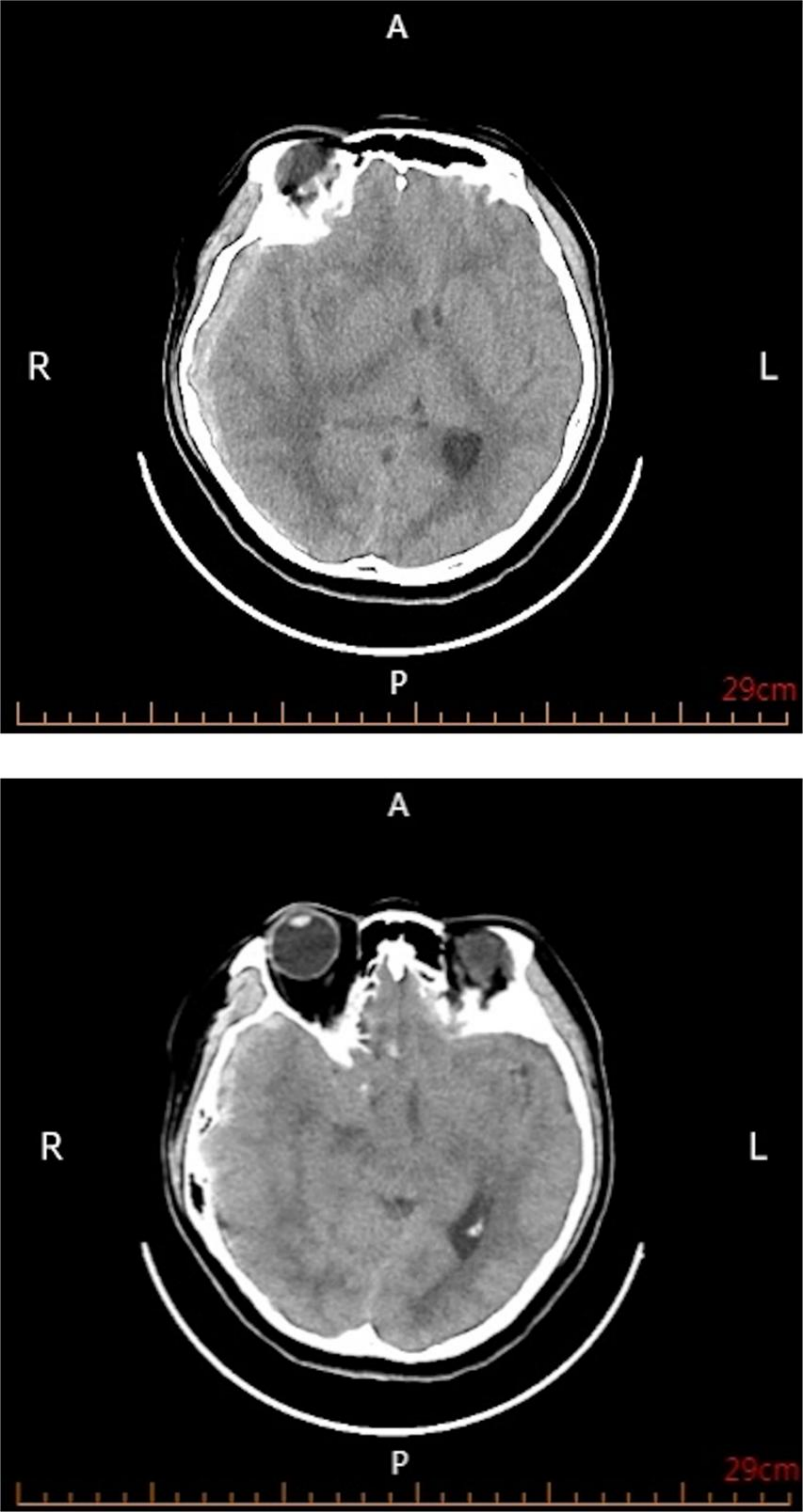
Preoperative non-contrast head CT. R, right; L, left; A, anterior; P, posterior.

**Figure 2:**
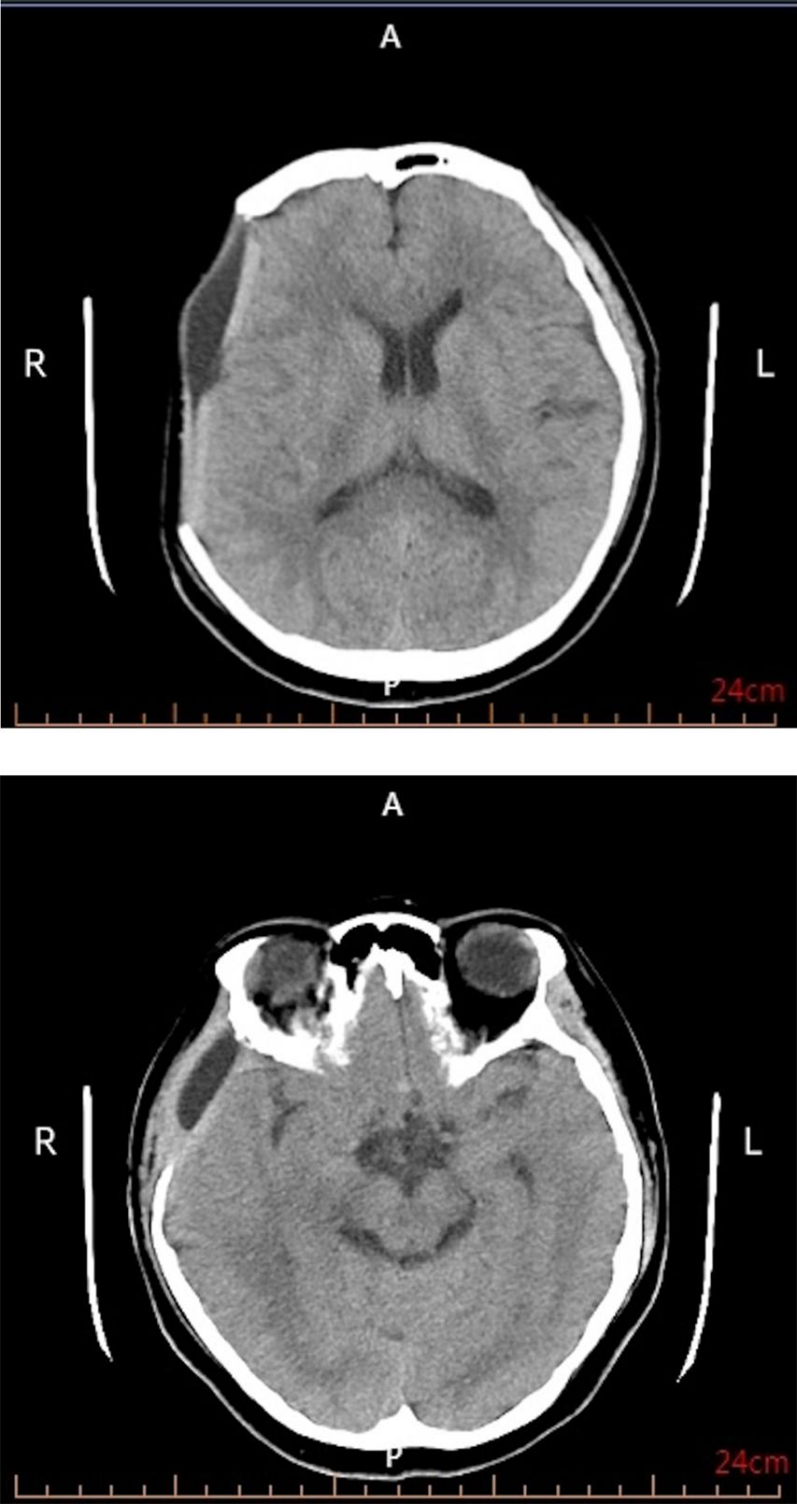
Postoperative follow-up CT. R, right; L, left; A, anterior; P, posterior.

She arrived hospital at 02:10 on May 13, 2024. Vital signs were temperature 38.3 °C, weight 78 kg, heart rate 110 beats/min, and blood pressure 177/93 mmHg. Head computed tomography (CT) demonstrated a large right fronto-temporo-parietal acute subdural hematoma with marked midline shift (Figure 1). This elevated blood pressure was interpreted as a stress response to acute intracranial pathology (sympathetic surge/Cushing response spectrum) rather than evidence of chronic hypertension, as she had no prior history of hypertension. Initial neurologic examination revealed a Glasgow Coma Scale (GCS) score of 10 (E3V3M4), isochoric pupils (2.5 mm) with sluggish light reflexes, and bilateral limb withdrawal corresponding to approximately grade II strength to painful stimulation (adopted Manual muscle strength testing, MMT). At 02:30, her neurologic status worsened to a GCS score of 9 (E2V3M4) with nuchal rigidity, while pupil size remained unchanged. Because of the rapid deterioration and the need to evaluate for a potential vascular etiology of spontaneous acute subdural hematoma, emergency digital subtraction angiography (DSA) was performed, which demonstrated no evidence of aneurysm, AVM, or dural arteriovenous fistula. These findings ruled out a vascular cause of the right-sided subdural hematoma, followed immediately by hematoma evacuation and decompressive craniectomy under general anesthesia (02:40). Two subcutaneous drains were placed, and invasive arterial blood pressure monitoring was established via a right radial arterial sheath. Shortly after ICU admission, invasive blood pressure decreased to 83/50 mmHg, requiring vasopressor support. Standard neurocritical care measures—including sedation, analgesia, osmotherapy, and anti-infective therapy—were initiated. Initial laboratory testing showed anemia (hemoglobin 120 g/L, normal range: 110–150 g/L) and provided the following coagulation and inflammatory profile: platelet count 176 × 10^9^/L (normal range: 100–300 × 10^9^/L), prothrombin time (PT) 13.5 s (normal range: 11.0–15.0 s) with international normalized ratio (INR) 1.04 (normal range: 0.80–1.20), activated partial thromboplastin time (aPTT) 32.0 s (normal range: 25.0–43.5 s), fibrinogen 3 g/L (normal range: 2–4 g/L), and D-dimer 8.74 ug/mL (normal range: 0.01–0.50 ug/mL). Inflammatory markers were C-reactive protein (CRP) 6 mg/L (normal range: 1–8 mg/L), and procalcitonin (PCT) 0.04 ng/mL. On May 14, with a stable postoperative appearance, she was extubated and transitioned to high-flow nasal cannula oxygen therapy, and one drain was removed. On May 16, she developed recurrent fever (peak 38.9 °C) and worsening anemia (hemoglobin 68 g/L). Repeat laboratory testing at clinical deterioration showed platelet count 139 × 10^9^/L, PT 14.1 s, INR 1.17, aPTT 35.7 s, fibrinogen 7 g/L, and D-dimer 0.97 ug/mL, along with inflammatory markers CRP 87 mg/L, and PCT 0.13. Given the combination of unexplained fever and progressive anemia without an alternative clear source on routine evaluation, a multidisciplinary discussion was held, and next-generation sequencing (NGS) was carried out. Follow-up CT on May 20 demonstrated postoperative changes after hematoma evacuation and decompressive craniectomy, with decreased compression of the right lateral ventricle and improved midline shift compared with the preoperative CT (Figures 2A,B). On May 23, blood NGS detected B19V. To corroborate systemic and potential central nervous system involvement, NGS was subsequently performed on a throat swab and cerebrospinal fluid, and B19V was also detected in both specimens. Based on these NGS findings, targeted parvovirus serologic testing was then ordered to support etiologic attribution; anti-parvovirus B19 IgM and IgG antibodies were positive. Supportive management included transfusion of 2 units of packed red blood cells (pRBCs) and intravenous immunoglobulin (IVIG). IVIG was discontinued on May 21 as the patient's clinical status stabilized. Hemoglobin improved to 92 g/L, the second drain was removed, fever resolved, and neurologic function progressively recovered, with limb strength improving to grade IV. She was transferred to a rehabilitation hospital on June 5 for ongoing therapy and recovery. A detailed clinical timeline of the episode of care is provided in Supplementary Table S1.

## Nursing

### Preoperative neurological assessment and care

Given the diagnosis of severe ASDH with intracranial hypertension and marked mass effect, the patient faced an imminent risk of cerebral herniation and brainstem compression. To interrupt the malignant cycle of secondary brain injury and reduced cerebral perfusion, a specialized neurocritical care protocol was immediately implemented.

Continuous multiparameter monitoring was initiated to track dynamic physiological changes. Systolic blood pressure (SBP) was strictly controlled between 110 and 130 mmHg to minimize the risk of hematoma expansion. Because fluctuations in vital signs often mirror intracranial dynamics, close observation was maintained for signs of Cushing's triad (e.g., bradycardia) and medullary dysfunction (e.g., Cheyne–Stokes respiration). Normothermia (<37.0 °C) was maintained to reduce cerebral metabolic demand.

**Figure 3:**
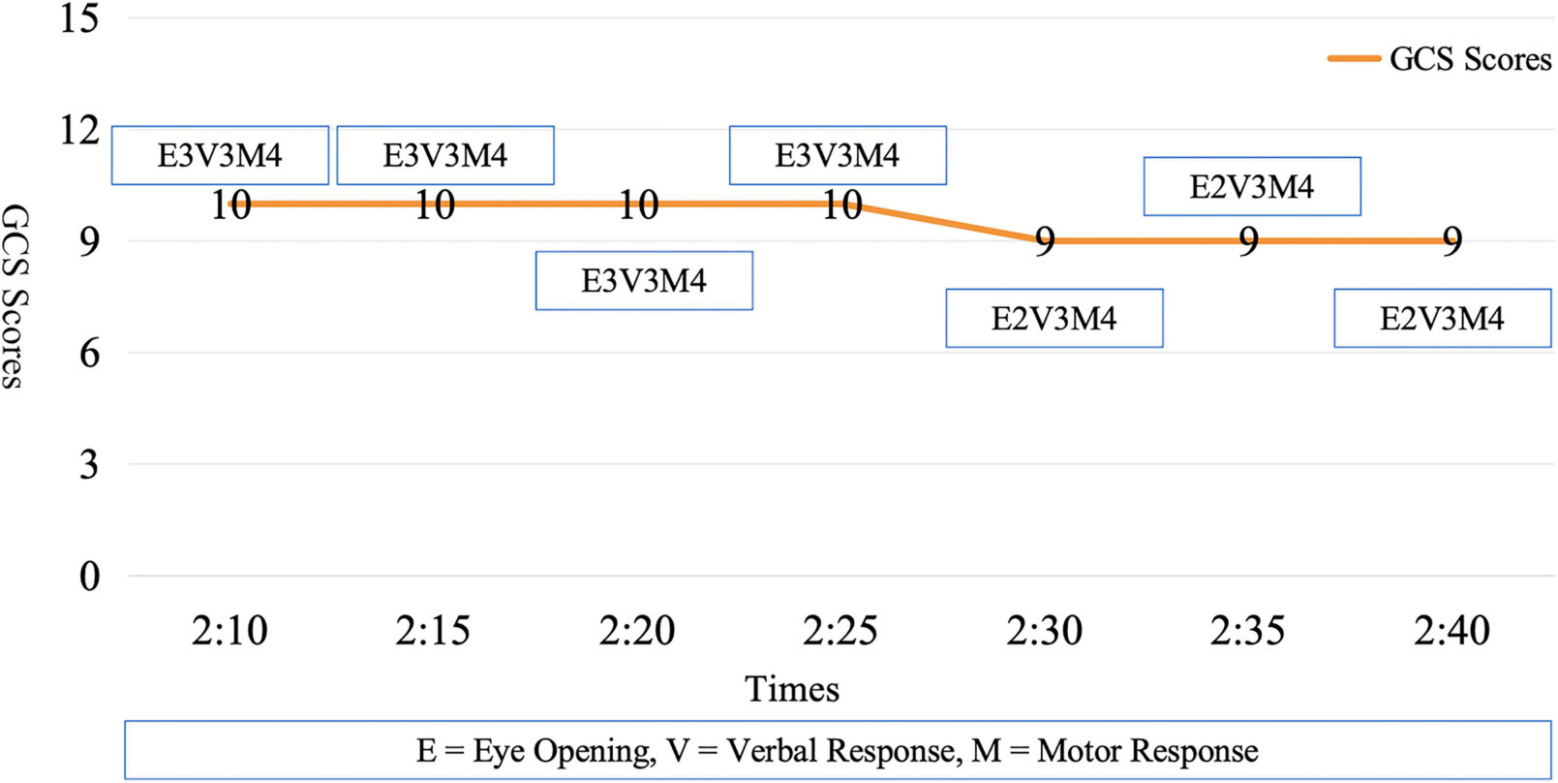
The monitoring trend of GCS.

**Figure 4:**
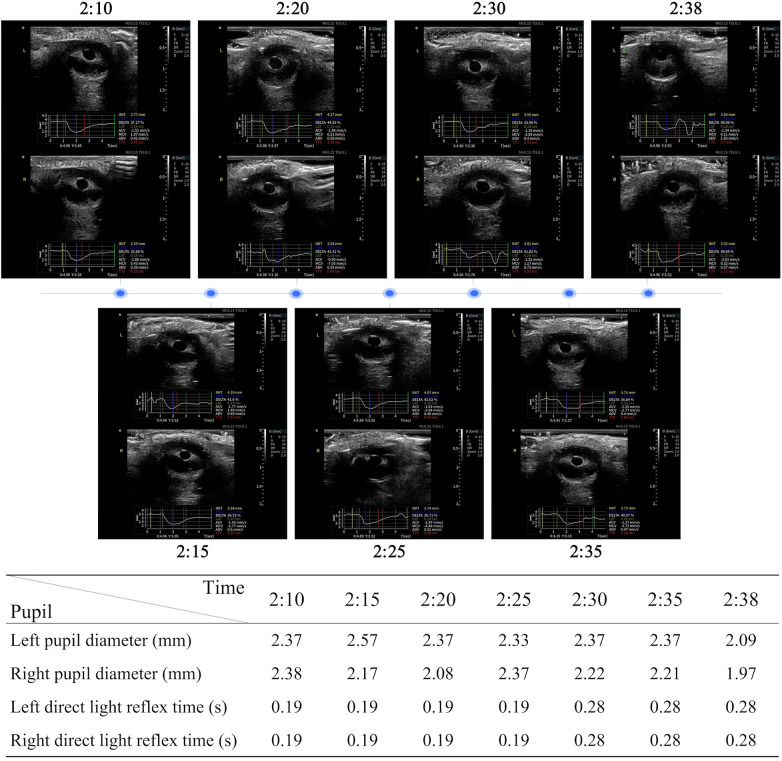
Preoperative pupil images of patients obtained using an automated ultrasonic pupil assessment device to determine pupil diameter and light reaction time.

Consciousness and motor function were assessed every 5–10 min. The patient's condition deteriorated, with the GCS score dropping from 10 to 9 (Figure 3). While conventional manual pupillary examinations revealed no obvious changes in pupil diameter or light reactivity, using an ultrasound-based pupil assessment device (ultrasound brand/model: Acclarix AX85), automated quantitative pupillometry detected subclinical progression: the light reflex latency for both pupils increased from 0.19 to 0.28s. These objective data provided an early warning of rising intracranial pressure before overt herniation, guiding timely clinical decision-making (Figure 4).

To facilitate venous drainage and reduce ICP, the head of the bed was elevated to 30°, with the head maintained in a neutral midline position. A quiet environment was enforced to minimize external stimuli that could trigger transient ICP spikes.

Osmotherapy was administered as prescribed to lower ICP, and an indwelling urinary catheter was placed for strict fluid balance monitoring. In parallel, rapid preoperative preparation was coordinated to facilitate immediate surgical intervention.

### Postoperative neurocritical management of B19V-associated ASDH

After surgery, the patient returned to the NICU in a critical state with multiple invasive devices (endotracheal tube, central venous catheter, two subcutaneous drains, urinary catheter) and a compressed right femoral puncture site following cerebral angiography. She remained hemodynamically unstable on vasopressors and was at high risk of both postoperative rebleeding and surgery-related thrombosis.

Positioning was strictly managed given the large right fronto-temporo-parietal bone window. A suspended hollow pressure-relief pad was used to avoid compression of the decompression site in the right lateral position. Nurses closely monitored scalp tension, color, perfusion, and the degree of brain bulging to detect early signs of swelling or herniation. The femoral puncture limb was immobilized, and peripheral circulation was monitored to prevent bleeding or ischemia.

Continuous invasive arterial blood pressure monitoring via a radial arterial sheath allowed real-time titration of vasopressor infusions to maintain adequate cerebral perfusion while minimizing peripheral ischemia. All lines were securely fixed, and hourly assessments of drainage output, neurological status (consciousness, pupils, limb strength), and vital signs were performed to detect rebleeding or catheter-related complications. To prevent venous thromboembolism during immobilization, the patient wore graduated compression stockings and performed ankle pump exercises based on the “Four-Level Rehabilitation Plan for Neurocritical Patients,” with regular monitoring of coagulation parameters. Pressure injury risk was evaluated using the Braden Scale, with repositioning every 2 h and pressure relief at bony prominences. No rebleeding, catheter-associated infections, thrombotic events, or pressure injuries occurred during the NICU stay.

### Infection control nursing for B19V

The patient's B19V infection was complicated by recurrent fever, anemia, and hypovolemic shock. Because B19V is transmitted via respiratory secretions and blood exposure, infection control focused on both patient safety and prevention of nosocomial spread. Core temperature and urine output were continuously monitored using a temperature-sensing urinary catheter connected to a closed anti-reflux system, placed under aseptic conditions to minimize catheter-associated infection. Given the lack of specific antiviral therapy beyond IVIG and blood transfusion (8), transfusions were performed under strict aseptic technique with close monitoring for transfusion reactions and IVIG-related nephrotoxicity. Renal function (including creatinine-based indices), hemoglobin levels, and inflammatory markers were followed dynamically. The patient was cared for in a single isolation room, and non-essential external examinations were minimized. Healthcare workers adhered to standard precautions and wore N95 respirators, caps, gloves, and gowns when entering the room, with rigorous hand hygiene. Environmental and terminal disinfection were reinforced using chlorine-based surface cleaning combined with ultraviolet irradiation, in accordance with institutional protocols.

### Implementation of ultra-early rehabilitation training

Ultra-early neurocritical rehabilitation aims to reduce complications of immobilization—such as deep vein thrombosis, ICU-acquired weakness, and delirium—and to promote functional recovery (15). In this case, rehabilitation nursing was initiated once vital signs and intracranial status were stabilized, following the “Four-Level Rehabilitation Plan for Neurocritical Patients” (16).

During deep sedation, Richmond Agitation-Sedation Scale: −4 (RASS −4), level-1 activities included regular turning, lower limb circulatory pump exercises, and passive limb mobilization, sometimes assisted by a passive cycling device. After extubation and recovery of consciousness, the patient progressed to level-2 and level-3 activities as tolerated, including bedside sitting and graded mobilization to maintain muscle strength and prevent lower limb deep vein thrombosis. Generally, the transition from Level 1 to Level 2 marks a significant shift in patient rehabilitation, moving from unconscious, passive reception to conscious, active participation in rehabilitative activities. Respiratory training (such as balloon-blowing exercises) accompanied weaning from humidified high-flow oxygen to low-flow nasal cannula. Swallowing function was screened at the bedside and found to be intact, allowing gradual advancement from a semi-liquid to a regular diet under close monitoring. Psychological care focused on orientation and emotional support, using family voice recordings or preferred music during the day and arranging daily video calls via the bedside communication system. Throughout hospitalization, the patient did not develop venous thrombosis, aspiration, ICU delirium, or pneumonia, supporting the feasibility and safety of ultra-early rehabilitation in this complex neurocritical setting.

## Discussion

This case illustrates an exceptionally rare presentation of acute subdural hematoma associated with acute human parvovirus B19 (B19V) infection in an otherwise healthy young adult. While B19V typically causes mild febrile illness, severe neurological complications are rarely reported in adults (14). In this patient, B19V-related erythropoietic suppression manifested as progressive anemia, and potential immune-mediated vascular injury may have increased vascular vulnerability, thereby contributing to the development and progression of intracranial hemorrhage (17). This observation highlights the importance of considering uncommon infectious etiologies when acute subdural hematoma occurs in the absence of trauma, vascular malformations, or coagulation disorders.

Although B19V-associated intracranial hemorrhage is exceedingly uncommon, viral infections as a broader category have been increasingly recognized as potential contributors to spontaneous intracerebral and subdural bleeding. Reported mechanisms include virus-induced thrombocytopenia, coagulation dysfunction, immune-mediated vasculitis, endothelial injury, and, in some cases, direct central nervous system invasion. Dengue virus is one of the most well-documented pathogens linked to intracranial hemorrhage, primarily through severe thrombocytopenia and increased vascular permeability, occasionally resulting in life-threatening hemorrhagic complications (18, 19). Herpes simplex virus (HSV) and varicella-zoster virus (VZV) have also been associated with hemorrhagic encephalitis and intracranial bleeding, most often in the context of viral vasculitis and necrotizing inflammation of cerebral vessels (20, 21). In immunocompromised populations, HIV infection has been linked to spontaneous intracranial hemorrhage related to vasculopathy, coagulopathy, or secondary opportunistic infections (22). More recently, SARS-CoV-2 infection has been associated with both ischemic and hemorrhagic cerebrovascular events, potentially mediated by endothelial dysfunction, systemic inflammation, and dysregulated coagulation (23, 24). Compared with these viral pathogens, B19V appears to differ in both epidemiology and pathophysiology. B19V-associated intracranial hemorrhage has been reported predominantly in fetuses or pediatric patients, whereas adult cases remain exceptionally rare. The proposed mechanisms involve suppression of erythropoiesis leading to anemia, immune-mediated vascular injury, and possible alterations in cerebral vascular integrity, rather than profound thrombocytopenia or overt coagulopathy. Recognition of this broader spectrum of virus-related intracranial hemorrhage provides important context for understanding both the rarity and the clinical significance of the present case.

The patient's rapid neurological deterioration emphasized the need for timely recognition of worsening intracranial hypertension. Preoperative neurocritical nursing played a central role in timely decision-making. Notably, while manual pupil checks did not reveal obvious changes, automated quantitative pupillometry detected subclinical latency prolongation (0.19–0.28s), providing an objective early warning of intracranial pressure elevation (25). These data, combined with strict blood pressure control, guided the decision for emergency hematoma evacuation, which successfully stabilized intracranial dynamics.

Postoperatively, the coexistence of hemodynamic instability, risk of rebleeding, and B19V-related anemia created additional complexity. Integrated neurocritical nursing measures—including invasive hemodynamic monitoring, careful management of drainage output, targeted droplet/contact precautions, and IVIG-related renal surveillance—effectively prevented secondary complications. The patient's clinical course also highlights the value of structured ultra-early rehabilitation, which contributed to functional recovery without inducing adverse events.

Overall, this case reinforces several key principles: (1) B19V infection should be considered as a potential contributor to hematological instability in neurocritical cases; (2) advanced monitoring tools like pupillometry can detect deterioration earlier than standard exams; and (3) individualized postoperative nursing and rehabilitation plans are crucial for optimizing outcomes in complex ASDH presentations.

## Conclusion

This rare case of B19V-associated acute subdural hematoma demonstrates the importance of multidisciplinary collaboration in neurocritical care. Comprehensive management—including precision diagnostics (automated pupillometry), timely surgical evacuation, targeted infection control, and structured ultra-early rehabilitation—enabled a favorable functional recovery. Clinicians should remain vigilant for viral etiologies in patients presenting with unexplained anemia or neurological deterioration. Further study is warranted to clarify the relationship between B19V infection and intracranial hemorrhage and to refine nursing strategies for similarly complex cases.

## Patient perspective

The patient manifested a high rate of satisfaction with the multidisciplinary neurocritical care, despite the complexity of the strict isolation protocols and intensive monitoring required for B19V infection. The satisfaction was mainly driven by the favorable functional recovery facilitated by ultra-early rehabilitation, including the rapid return of consciousness and improvement in limb strength.

## Data Availability

The original contributions presented in the study are included in the article/Supplementary Material, further inquiries can be directed to the corresponding author.
