# Supplementary material for: Case Report: Life-threatening acute subdural hematoma associated with human parvovirus B19 infection in a young adult
Source: Front Surg. 2026 Feb 25;13:1762977. doi: 10.3389/fsurg.2026.1762977 (PMC12977093; doi:10.3389/fsurg.2026.1762977)
Supplement: Supplementary file 1 [file Table1.docx]

**Supplementary Table 1**

**Clinical Timeline: HPVB19-Related Acute Subdural Hamatoma Case**

| Time (relative) | Date / Time | Key events & clinical status | Major investigations & interventions |
| --- | --- | --- | --- |
| 7 days before admission | Approx. 1 week before 13 May 2024 | Travel to Guangzhou with her children approximately 1 week before symptom onset; two of her children developed fever three days before admission, while the patient herself remained clinically well and did not seek medical attention. | No medical consultation was sought; after admission, acute human parvovirus B19 infection was confirmed based on positive serum anti–B19 IgM and IgG antibodies. |
| Day 0 | 13 May 2024, 02:10 | ED arrival with sudden severe headache and dizziness, fever (38.3°C); GCS 10 (E3V3M4); pupils ~2.5 mm with sluggish light response; bilateral limb strength II. | Emergency non-contrast head CT: large right fronto-temporo-parietal acute subdural haematoma with significant midline shift; emergency neurosurgical consult arranged. |
| Day 0 (pre-op) | 13 May 2024 (pre-surgery) | Rapid neurological deterioration: GCS falls from 10 to 9 with progressive neck stiffness; manual pupil size unchanged, but clinical concern for rising intracranial pressure. | Quantitative pupillometry shows prolonged light-reflex latency compared with baseline, indicating early pupillary deterioration; decision made for urgent craniotomy and decompression. |
| Day 0 (surgery) | 13 May 2024, ~02:40–07:33 | Critically ill status requiring emergency surgery under general anaesthesia; patient remains intubated and sedated throughout the procedure. | Emergency cerebral angiography; right fronto-temporo-parietal haematoma evacuation with right partial decompressive craniectomy; placement of drains and invasive lines; postoperative transfer intubated to the neuro-ICU. |
| Day 0–1 | 13–14 May 2024 | Postoperative neurocritical care with mechanical ventilation, deep sedation, and analgesia; persistent fever and haemodynamic instability requiring vasopressors and close monitoring. | Invasive arterial blood pressure monitoring via a radial arterial line; strict blood pressure management, osmotherapy, and empirical anti‑infective therapy; initiation of level‑1 rehabilitation nursing (regular turning, limb positioning, and passive range‑of‑motion exercises) as tolerated. |
| Day 1 | 14 May 2024 | Neurological status improves; patient becomes more responsive; readiness for weaning from ventilation; no signs of recurrent intracranial hypertension. | Follow-up head CT confirms satisfactory haematoma evacuation and no rebleeding; one subgaleal drain removed; endotracheal tube removed and patient switched to high-flow nasal cannula; continuation of structured early neurorehabilitation. |
| Day 3 | 16 May 2024 | Recurrent high fever up to 38.9°C; haemoglobin drops to 68 g/L, consistent with anaemia and hypovolaemic shock related to acute B19V infection. | Laboratory tests confirm marked anaemia and ongoing B19V infection; supportive management includes transfusion of 2 units of packed red blood cells and initiation of intravenous immunoglobulin (IVIG), with close haemodynamic and temperature monitoring. |
| Days 4–7 | 17–20 May 2024 | Fever resolves; haemodynamics gradually stabilise; consciousness and limb strength improve, allowing progressive bedside sitting and participation in rehabilitation activities with assistance. | Ongoing IVIG therapy and serial laboratory monitoring; gradual upgrade of the rehabilitation programme, including respiratory training, limb strengthening exercises, and swallowing assessment and practice under multidisciplinary supervision. |
| Day 8 | 21 May 2024 | Clinically stable and afebrile; clear consciousness; bilateral limb muscle strength around grade IV; able to participate in active rehabilitation exercises. | IVIG discontinued as the patient’s condition stabilises; haemoglobin rises to approximately 92 g/L and remains stable; the second subcutaneous drainage tube is removed; continuation of comprehensive rehabilitation in the neurosurgical unit. |
| Day 23 | 5 June 2024 | Neurologically stable with clear consciousness and bilateral limb muscle strength at approximately grade IV. | Transferred to a rehabilitation hospital for continued rehabilitation care. |
